# Dual-responsive nanoplatform for integrated cancer diagnosis and therapy: Unleashing the power of tumor microenvironment

**DOI:** 10.3389/fchem.2024.1475131

**Published:** 2024-09-26

**Authors:** Rui Ma, Peng Zhang, Xiuying Chen, Mengdi Zhang, Qinghe Han, Qinghai Yuan

**Affiliations:** Department of Radiology, The Second Hospital of Jilin University, Changchun, China

**Keywords:** metal-polyphenol network, computed tomography, magnetic resonance imaging, chemodynamic therapy, photothermal therapy

## Abstract

Chemodynamic therapy (CDT), designed to trigger a tumor-specific hydrogen peroxide (H_2_O_2_) reaction generating highly toxic hydroxyl radicals (·OH), has been investigated for cancer treatment. Unfortunately, the limited Fenton or Fenton-like reaction rate and the significant impact of excessive reducing glutathione (GSH) in the tumor microenvironment (TME) have severely compromised the effectiveness of CDT. To address this issue, we designed a dual-responsive nanoplatform utilizing a metal-polyphenol network (MPN) -coated multi-caged IrO_x_ for efficient anti-tumor therapy in response to the acidic TME and intracellular excess of GSH, in which MPN composed of Fe^3+^ and tannic acid (TA). Initially, the acidic TME and intracellular excess of GSH lead to the degradation of the MPN shell, resulting in the release of Fe^3+^ and exposure of the IrO_x_ core, facilitating the efficient dual-pathway CDT. Subsequently, the nanoplatform can mitigate the attenuation of CDT by consuming the excessive GSH within the tumor. Finally, the multi-caged structure of IrO_x_ is advantageous for effectively implementing photothermal therapy (PTT) in coordination with CDT, further enhancing the therapeutic efficacy of tumors. Moreover, the outstanding Computed Tomography (CT) and Magnetic Resonance Imaging (MRI) (T_1_/T_2_) multimodal imaging capabilities of IrO_x_@MPN enable early diagnosis and timely treatment. This work provides a typical example of the construction of a novel multifunctional platform for dual-responsive treatment of tumors.

## 1 Introduction

CDT utilizes the endogenous H_2_O_2_ that existed in the TME to produce· OH (Liu et al., 2023; Zou et al., 2022; Lin et al., 2020). Fenton or Fenton-like reactions encompass a class of processes where Fe^3+^, iron-containing minerals, and other transition metal ions can either accelerate or substitute for Fe^2+^ in catalyzing the decomposition of H_2_O_2_ (Jana and Zhao, 2022; Zhang et al., 2022; Zheng et al., 2022; Getachew et al., 2021). The ·OH formed can compromise tumor cells by causing lipid peroxidation, inactivating proteins, and inflicting DNA damage, which in turn induces oxidative stress and promotes cell death (Meng et al., 2020). Unlike other treatment approaches, CDT is specifically triggered by internal biological signals, which enhances its selectivity and makes it a highly targeted therapeutic strategy (Tang et al., 2021). Additionally, it exhibits high sensitivity to various factors in the TME, including catalytic ion concentration and multiple factors such as H₂O₂, GSH, and pH values. Despite its considerable promise, CDT as a sole therapeutic approach faces limitations due to the scarcity of endogenous H_2_O_2_ in the tumor environment and the decreased effectiveness of Fenton or Fenton-like reactions in the mildly acidic pH range of 5.6–6.8. Simultaneously, the GSH-mediated cellular antioxidant defense system (ADS) can eliminate reactive oxygen species (ROS) within cells, significantly reducing the ·OH production rate and the therapeutic efficiency of CDT (Lin et al., 2019; Wang Z. et al., 2019; Hao et al., 2020). Therefore, depleting GSH to overcome the protective effect of ADS is crucial for enhancing the CDT effect. Moreover, achieving optimal therapeutic results with CDT alone is challenging due to the complexity, diversity, and variability within the TME. Currently, combined cancer therapy has emerged and undergone in-depth research, significantly improving treatment effectiveness while minimizing adverse reactions (Jiang et al., 2021; Manivasagan et al., 2022; Feng et al., 2021). Among various combination cancer treatments, the pairing of PTT with CDT stands out as a highly promising approach, offering superior selectivity, reduced toxicity, and minimal invasiveness (Manivasagan et al., 2022; Feng et al., 2021). PTT harnesses light absorption by photothermal agents to generate localized heat or induce thermal ablation, effectively targeting and destroying cancer cells (Li et al., 2020). Research has shown that increasing the temperature within the tumor site can notably improve the effectiveness of Fenton or Fenton-like reactions, thus accelerating the generation of ·OH radicals (Wang et al., 2020; Wang X. et al., 2019). Therefore, the exploration of a highly efficient nanoplatform for combined CDT/PTT to maximize the antitumor effect represents an attractive research direction (Liu et al., 2019; Huang et al., 2017; Abbas et al., 2017; Liu et al., 2018).

The nanoplatforms composed of iridium (Ir) have sparked widespread interest among researchers across various fields due to their unique optical and physical properties (Yin et al., 2021; Shen et al., 2021). On one hand, it has been shown to exhibit robust catalase activity for oxygen evolution across a diverse range of pH levels (An and Tc, 2002; Yip and Lo, 2018). On the other hand, Ir-based complexes are distinguished by their superior photothermal conversion capabilities, impressive photostability, and low levels of cytotoxicity (Zhen et al., 2020a; Zhen et al., 2020b; Zhen et al., 2018). Furthermore, due to the significant atomic number (Z = 77) and robust nature of the transition metal Ir element, IrO_x_ exhibits excellent X-ray absorption capability and can be employed for CT imaging (Zhen et al., 2020b). Based on the characteristics mentioned above, Ir-based nanomaterials could be employed for CDT and PTT of tumors, achieving promising therapeutic outcomes (Deng et al., 2022; Nie et al., 2022). While Ir-based nanomaterials hold tremendous potential, their standalone catalysis in initiating Fenton-like reactions is limited and cannot overcome the constraints of the ADS system. Therefore, it is necessary to explore functionalized Ir-based nanomaterials to meet these requirements.

Herein, we developed a core-shell nanoplatform (IrO_x_@MPN) that is responsive to both the acidic TME and GSH for combined CDT/PTT and CT/MRI imaging of tumors. This nanoplatform based on IrO_x_ is coated by the metal-polyphenol network (MPN) and features Fe^3+^ and tannic acid (TA) coating on multi-caged IrO_x_ nanoparticles, to achieve integrated diagnosis and treatment. MPN, consisting of Fe^3+^ and tannic acid (TA), possesses pH-responsiveness, as polyphenols readily dissociate from metal ions in low pH environments (Liu et al., 2023), allowing for controlled release of Fe^3+^. Moreover, MPN could dissociate under intracellular stimuli, such as adenosine triphosphate (ATP) and GSH (Peng et al., 2022), thus exposing the IrO_x_ core. The IrO_x_ core exhibits intrinsic activities similar to catalase and peroxidase, enabling the conversion of excessive H_2_O_2_ in the tumor tissue into oxygen (O_2_) and generating ·OH. Moreover, the self-cyclic valence alternation between Ir^4+^ and Ir^3+^ allows IrO_x_ to continuously consume GSH, alleviating tumor hypoxia and improving antioxidant capacity. Simultaneously, Fe^3+^ can be reduced to Fe^2+^ by TA and GSH, and Fe^2+^ exhibits catalytic performance, reacting with the H_2_O_2_ presented in the TME to generate ·OH through Fenton reactions. The released Fe^3+^ can further deplete GSH, enhancing the CDT effect. The multi-caged structure of IrO_x_ could enhance the photothermal effect and loading capacity (Zhen et al., 2018), resulting in high photothermal conversion efficiency (60.78%). Due to the coordination of polyphenols with Fe^3+^ ions in MPN (Liu et al., 2023; Cen et al., 2023; Wang et al., 2022; Spinello et al., 2016; Guo et al., 2014), the nanoplatform also possesses a pH-activated enhancement of T_1_-T_2_ dual-modal imaging capability in the TME. Additionally, the IrO_x_ core exhibited excellent CT imaging capability. Therefore, IrO_x_@MPN represents a promising dual-responsive CDT/PTT multifunctional nanoplatform with concurrent CT and MRI multimodal imaging capabilities.

## 2 Materials and methods

### 2.1 Materials

Iridium trichloride (99.99%, metal basis), 1,3-diphenylisobenzofuran (DPBF), Methylene blue (MB), 1,10-phenanthroline, sodium hydroxide (NaOH), calcein acetoxymethyl ester (Calcein AM), propidium iodide (PI) was sourced from Aladdin Reagent Co. (Shanghai, China). 5, 50-dithiobis (2-nitrobenzoic acid) (DTNB), ethanol, hydrogen peroxide (H_2_O_2_, 30%), [Ru (dpp)_3_]Cl_2_ (RuDPP), 2,7-dichlorofluorescin diacetate (DCFH-DA), Fluorescein Isothiocyanate Isomer I (FITC), dimethyl sulfoxide (DMSO) was obtained from Sigma-Aldrich (America). Hematoxylin-eosin (H&E), and Dulbecco’s modified eagle’s medium (DMEM) were acquired from Thermo Scientific Co. (Beijing, China). Throughout the experiments, deionized (D.I.) water was used exclusively. All chemicals were used directly as provided, without any additional purification steps.

### 2.2 Characterizations

Transmission electron microscopy imaging was performed with FEI TECNAI G2 F20. The UV-visible absorption spectra were recorded by Hitachi U-3100 spectrophotometer. X-ray photoelectron spectroscopy was conducted with an ESCALab220i-XL electron spectrometer. The binding energies for all analyzed elements were referenced to the C 1s peak of adventitious carbon, with the energy fixed at 284.6 eV. The content of Ir and Fe elements was determined using ICP-OES (Thermo Fisher Scientific, United States). The dissolved oxygen content was assessed with a portable dissolved oxygen meter (JPBJ-608) from Shanghai Instrument Scientific Instrument Co., Ltd., China. Fluorescence images of cells were captured with a Leica TCS SP2 confocal laser scanning microscope. The pH value was obtained using a pH meter (PB-10) from Sartorius, Germany.

### 2.3 Preparation of multi-caged IrO_x_

IrCl_3_·6H_2_O (0.1493 g) was dissolved in 50 mL of water and allowed to be stirred for 3 h. The solution was then kept at 4°C for about 3 days until it cleared. Then, the IrCl_3_ aqueous solution was kept at room temperature for ∼2 h. Next, a 1.0 M NaOH solution was slowly added to bring the pH to 12. The mixture was then continuously stirred at room temperature for an additional 2.5 h. Afterward, it was transferred to an oil bath, where vigorous stirring was maintained as the temperature was raised to 80°C. The oil temperature could rise to 80°C within 25 min. The reaction was continued for 13 h under conditions that facilitated condensation. The IrO_x_ nanoparticles were subsequently isolated by spinning at 15,000 rpm for 10 min, then thoroughly washed with ethanol and water before being prepared for further use. The collected nanoparticles were freeze-dried and stored for later applications.

### 2.4 Preparation of multi-caged IrO_x_@MPN

IrO_x_ NPs (8 mL, 4 mM) were placed into a 10 mL centrifuge tube. 40 μL of TA (40.8 mg/mL) was added to the IrO_x_ NPs for 20 s. Next, 40 μL of FeCl_3_ (48 mM) was added. Next, 2 mL of Tris buffer (50 mM, pH 8.0) was introduced, followed by blending the mixture for 20 s. The solution was subsequently spun at 12,000 rpm for 10 min, followed by two washings with ddH_2_O to isolate IrO_x_@MPN. The concentrations of Ir and Fe in the prepared IrO_x_@MPN were measured to be 307.3 and 6.7 μg/mL by ICP-OES, respectively. IrO_x_@MPN with different Ir/Fe ratios were synthesized using the same procedure, with the exception of altering the concentration of FeCl_3_.

### 2.5 Preparation of Fluorescein Isothiocyanate Isomer I-conjugated IrO_x_@MPN (IrO_x_@MPN-FITC)

To investigate the intracellular uptake of IrO_x_@MPN, Fluorescein Isothiocyanate Isomer I (FITC)-loaded IrO_x_@MPN was prepared. Firstly, 500 μg of FITC was combined with 1 mL of dimethyl sulfoxide (DMSO) and mixed at room temperature for 1 h. Next, 1 mL of IrO_x_@MPN solution in deionized water (2.5 mg/mL) was introduced and stirred overnight. The product was thoroughly rinsed with deionized water until the supernatant was almost completely clear.

### 2.6 Extracellular Fe^2+^ generation

Reduced Fe^2+^ was detected using 1,10-phenanthrolin. 1 mg of IrO_x_@MPN, with or without GSH (10 mM), was suspended in 1 mL of aqueous solution at pH 7.4 or 5.0 and stirred for 1 h. Subsequently, 1 mL of 1,10-phenanthroline ethanol solution (1 mg/mL) was combined with 1 mL of IrO_x_@MPN aqueous solution, with or without GSH, and the mixture was left to sit for 10 min. Absorbance was then assessed at 508 nm with UV-Vis spectrophotometry. For comparison, UV-Vis spectra of 1,10-phenanthroline alone were also recorded.

### 2.7 Extracellular O_2_ generation

Oxygen generation was monitored with a dissolved oxygen (DO) meter. Generally, 29.7 mL of IrO_x_@MPN solution (Ir concentrations: 0.5 mM) was combined with 300 μL of H_2_O_2_ solution (100 μM) while stirring vigorously. The changes in dissolved oxygen were automatically recorded at 10s intervals. All the solutions were pre-saturated with N_2_ until the dissolved oxygen was 0%. As controls, the generated O_2_ of H_2_O, H_2_O_2_, and IrO_x_/H_2_O_2_, were also monitored for comparison.

### 2.8 Extracellular ·OH generation

·OH was detected using methylene blue (MB). Firstly, IrO_x_@MPN (Ir concentration: 100 μg/mL) was incubated with aqueous solutions (pH = 7.4 or 5.0) with or without GSH (1 mM) for 3 h. Next, MB aqueous solution (3 mL, 10 mg/L), H_2_O_2_ (100 μM), and IrO_x_@MPN aqueous solutions were thoroughly mixed, followed by centrifugation to collect the supernatant. Absorption around 660 nm was subsequently measured using UV-Vis spectroscopy. The variations in MB absorption in different groups were similarly assessed following the same procedures, including MB, MB/H_2_O_2_, MB/IrO_x_, MB/IrO_x_@MPN, MB/IrO_x_@MPN/GSH, MB/IrO_x_@MPN/H^+^, MB/IrO_x_@MPN/H_2_O_2_, MB/IrO_x_@MPN/GSH/H_2_O_2_, MB/IrO_x_@MPN/H^+^/H_2_O_2_, and MB/IrO_x_@MPN/H^+^/GSH/H_2_O_2_.

### 2.9 Extracellular GSH depletion

The GSH level was investigated by utilizing 5, 50-dithiobis (2-nitrobenzoic acid) (DTNB). GSH aqueous solution (1 mM) was added into IrO_x_ and IrO_x_@MPN aqueous solution (50 μg/mL). DTNB PBS solution (1.5 mg/mL) was added into IrO_x_@MPN aqueous solution after different reaction times, and the absorbance of the mixture was then analyzed using a UV-Vis spectrophotometer.

### 2.10 Photothermal of IrO_x_@MPN

1 mL of IrO_x_@MPN PBS solutions with varying Ir concentrations (0, 0.125, 0.25, 0.5, 1 mg/mL) were subjected to an 808 nm laser with an intensity of 1 W/cm^2^ for 10 min, The temperature of these solutions was tracked using a thermocouple probe, and measurements were taken every 30 s. Additionally, to evaluate the thermal stability of IrO_x_@MPN, an aqueous solution of IrO_x_@MPN (0.5 mg/mL) was subjected to an 808 nm laser with an intensity of 1 W/cm^2^ for 10 min in a quartz cuvette, and the temperature was lowered down through natural cooling, repeated the above steps for five times.

### 2.11 Incubation of cells

Mouse breast cancer cells (4T1) and mouse fibroblasts (L929) were provided by the Institute of Biochemistry and Cell Biology, Chinese Academy of Sciences. 4T1 and L929 cells were grown in DMEM medium ([glucose] = 4.5 g/L) enriched with 10% fetal bovine serum (FBS), penicillin (100 units/mL), and streptomycin (100 μg/mL). The cells were plated at a concentration of 10^6^ cells per 25 cm^2^ flask and incubated at 37°C in a 5% CO₂ environment for 24 h.

### 2.12 *In Vitro* cytotoxicity study

The 96-well plates were seeded with L929 and 4T1 cells at a density of 5 × 10³ cells per well and incubated for 24 h. Each well received 100 μL of IrO_x_@MPN medium solutions with concentrations of 0, 13, 25, 50, 100, and 200 μg/mL, and was then incubated for an extra 24 h. Subsequently, the cells were rinsed three times with PBS, after which 100 μL of CCK-8 DMEM solution (CCK-8: DMEM = 1:10) was added. The cells were further cultured for 1 h. Cell viability was determined by analyzing the absorbance at 450 nm with a microplate reader.

### 2.13 Cellular uptake of IrO_x_@MPN

Cells of the 4T1 line were introduced into a 6-well culture plate and maintained in a 37°C environment with 5% CO_2_ for a duration of 24 h, Subsequently, the cells were exposed to IrO_x_@MPN-FITC for various durations, specifically 30 min, 1 h, 2 h, and 4 h. Then, the cells were subjected to two washes with PBS and then stained with Hoechst 33,342. The cellular uptake was observed using an intracellular fluorescence microscope.

### 2.14 Intracellular GSH consumption

Cells of the 4T1 line were introduced into a 6-well culture plate and maintained in a 37°C environment with 5% CO_2_ for a duration of 24 h. Then, the cells were further incubated with IrO_x_ and IrO_x_@MPN medium solutions (50 μg/mL) for 24 h. Subsequently, each well was rinsed with PBS three times, and the cells were harvested and centrifuged at 3,000 rpm for 10 min. Then, cells were disrupted by RIPA Lysis buffer for 5 min and re-suspended in 500 μL of PBS. An equal volume of 100 μL cell lysate and reagent 1) from the assay kit were mixed and subjected to centrifugation at 3,500 rpm for 10 min. Following centrifugation, 100 μL of the resulting supernatant was combined with 100 μL of reagent 2) and 25 μL of reagent 3). The absorbance at 420 nm was subsequently recorded using UV-Vis spectroscopy. A UV-Vis absorption spectrum of a GSH standard sample (20 μmol/L) was recorded for comparison.

### 2.15 Intracellular O_2_ generation

[(Ru (dpp)_3_)]Cl_2_ was employed as the O_2_ sensing probe. 4T1 cells were seeded into a 96-well plate and incubated under the same conditions as previously used for cell culture. Next, a 5 μM solution of [(Ru (dpp)_3_)]Cl_2_ was added to the cells and left for incubation over 4 h. Following this, the plate was washed with PBS and further incubated with DMEM medium, IrO_x_ (50 μg/mL in DMEM), IrO_x_@MPN (50 μg/mL in DMEM) for 4 h. Finally, after washing the cells with PBS, red fluorescence images were captured using an intracellular fluorescence microscope (λ_ex_ = 488 nm, λ_em_ = 620 nm).

### 2.16 Intracellular ROS production

We detected intracellular ROS using 2,7-dichlorofluorescin diacetate (DCFH-DA) as the probe. 4T1 cells were seeded into a 6-well plate and incubated under the same conditions as previously used for cell culture. Next, the cells were exposed to 10 μM DCFH-DA in DMEM for 20 min. After washing the wells three times with PBS, they were further incubated for 12 h with either 2 mL of DMEM alone, DMEM with 50 μg/mL IrO_x_, or DMEM with 50 μg/mL IrO_x_@MPN. After another round of washing with PBS, images were captured using an intracellular fluorescence microscope (λ_ex_ = 504 nm, λ_em_ = 529 nm).

### 2.17 *In Vitro* chemodynamic therapy

4T1 cells were seeded into a 96-well plate and incubated under the same conditions as previously used for cell culture. They were then treated with either DMEM, a 50 μg/mL IrO_x_ solution in DMEM, or a 50 μg/mL IrO_x_@MPN solution in DMEM for 12 h. Following treatment, cell viability was evaluated using an intracellular fluorescence microscope after a 30-min staining with Calcein AM and PI.

### 2.18 *In Vitro* photothermal therapy

After cultivating 4T1 cells in a 96-well plate, they were treated with DMEM, a 50 μg/mL IrO_x_ solution in DMEM, or a 50 μg/mL IrO_x_@MPN solution in DMEM for 12 h. Following a PBS wash, the 4T1 cells were exposed to 808 nm laser irradiation at 1.0 W/cm^2^ for 10 min. Cell viability was then evaluated using an intracellular fluorescence microscope after a 30 min staining with Calcein AM and PI. The efficiency of photothermal conversion (η) was determined using the equations outlined below.

$$\theta =\frac{{T-Tsurr}_{\left(sample\right)}}{T_{\max\left(sample\right)}-T_{surr\left(sample\right)}}$$

$$t=\tau_{s}\times \left(-In\theta \right)$$

$$hS=\frac{m_{d}\times c_{d}}{\tau_{s}}$$

$$Q_{dis}=hS\left(T_{\max\left(water\right)}-T_{surr\left(sample\right)}\right)$$

$$\eta =\frac{hS\left(T_{\max\left(sample\right)}-T_{surr\left(sample\right)}\right)-Q_{dis}}{I\left(1-10^{A808}\right)}$$

$$hS=\frac{m_{d}\times c_{d}}{\tau_{s}}$$

h (mWm^−2^ °C^−1^): Heat transfer coefficient.S (m^2^): Surface area of the container. T_max_ (°C): Equilibrium temperature. T_surr_ (°C): Ambient temperature of the surroundings. Q_Dis_ (mW): The heat from light absorbed by the quartz cuvette walls itself was measured independently using a quartz cuvette cell containing aqueous samples without NPs.

### 2.19 *In vivo* therapy

All the animal experiments were conducted following the National Regulation of China for Care and Use of Laboratory Animals and approved by the Institutional Animal Care and Use Committee of Changchun Institute of Applied Chemistry, Chinese Academy of Sciences (Grant no. 20230065). *In vivo*, therapy was conducted using a subcutaneous tumor model. Each mouse was injected subcutaneously with 100 μL of a suspension containing 4 × 10^6^ 4T1 cells into the right hind leg. The tumors were permitted to develop until they reached approximately 100 mm³ in size. Tumor models were established using female Balb/C mice aged 6–8 weeks, following the procedures described above. The mice were allocated into six distinct groups, each consisting of five mice (n = 5). These groups were: 1) control (saline solution injection); 2) Laser; 3) IrO_x_ (injected with a 100 μL solution of IrO_x_ at 10 mg/kg); 4) IrO_x_/Laser; 5) IrO_x_@MPN (injected with a 100 μL solution of IrO_x_@MPN at 10 mg/kg); 6) IrO_x_@MPN/Laser. After intravenous injection of 24 h, the tumors were subjected to 808 nm laser irradiation at 1 W/cm^2^ for 10 min. Every mice was injected every 2 days. Additionally, tumor dimensions were measured using a digital caliper, and the volume was calculated with the formula: *Volume = (Length × Width* ^*2*^ *)/2*. The relative tumor volume was determined by dividing the post-treatment volume *V* by the pre-treatment volume *V₀*. Concurrently, the mice’s body weight was tracked bi-daily to monitor any long-term toxic effects *in vivo*. On the 15th day, tumors from each treatment group were surgically removed and weighed. Finally, the mice’s major organs were excised and preserved in 4% paraformaldehyde for histological analysis. This procedure was aimed at further evaluating the biocompatibility of IrO_x_ and IrO_x_@MPN.

### 2.20 *In vitro* and *In vivo* CT imaging

CT images of IrO_x_@MPN solutions (Ir concentration: 0, 0.25, 0.5, 1, 2, 4, 8, and 16 mg/mL) were measured using CT Scanner. For *in vivo* CT imaging, the tumor-bearing mice were intravenously injected with 100 μL of IrO_x_@MPN (3 mg/mL in saline). CT scans were performed at different intervals post-injection, specifically at 0 h, 2 h, 24 h, 48 h, and 72 h. Coronal and 3D images were generated using multiplanar reconstruction.

### 2.21 *In vitro* and *In vivo* MRI imaging

MRI images of IrO_x_@MPN solutions were measured using IngFenia CX 3.0 T MR scanner, with a 32-channel body coil. MR sequences included T_1_-weighted imaging (T_1_WI), and T_2_-weighted imaging (T_2_WI). The T_1_ and T_2_ relaxation time was measured using IrO_x_@MPN solutions with different Fe concentrations by a workstation (IntelliSpace Portal, Philips Healthcare). To facilitate *in vivo* MRI imaging, once the tumors reached approximately 150 mm³, 100 μL of IrO_x_@MPN (2 mg/mL in saline) was administered intravenously to the mice, and imaged by the MR scanner, respectively after 0 h (pre-), 2 h, 24 h, 48 h of injection.

### 2.22 blood analysis

Eight mice were divided into two groups to assess blood parameters. In one group, four mice received an intravenous injection of IrO_x_@MPN at a dosage of 10 mg/kg. On the 30th day, blood samples were drawn from the eye sockets of these mice. Blood from untreated, healthy mice was used as a control.

### 2.23 Histology staining

On the 15th day of treatment, tumor-bearing mice were euthanized, and the tumors were removed, fixed in 10% neutral buffered formalin, and then embedded in paraffin. On the 30th day, the mice were re-dissected, and their tissues were similarly fixed in formalin and paraffin-embedded. Sections of tumors, each 4 μm thick, were stained with hematoxylin and eosin (H&E) and subjected to terminal deoxynucleotidyl transferase-mediated nick end labeling (TUNEL) staining. Normal organs were also sliced into 4 μm sections for H&E staining.

### 2.24 Statistical analysis

Each experiment was repeated a minimum of three times, and the results are presented as mean ± S.D. To determine significant differences between groups, one-way ANOVA followed by Dunnett’s post-hoc test was used. Statistical significance was assumed at a value of ****p < 0.0001, ***p < 0.001, **p < 0.01, *p < 0.1, and ns: no significant difference. All data were analyzed using Origin 8.0 and Excel 2016.

## 3 Results and discussion

**FIGURE 1:**
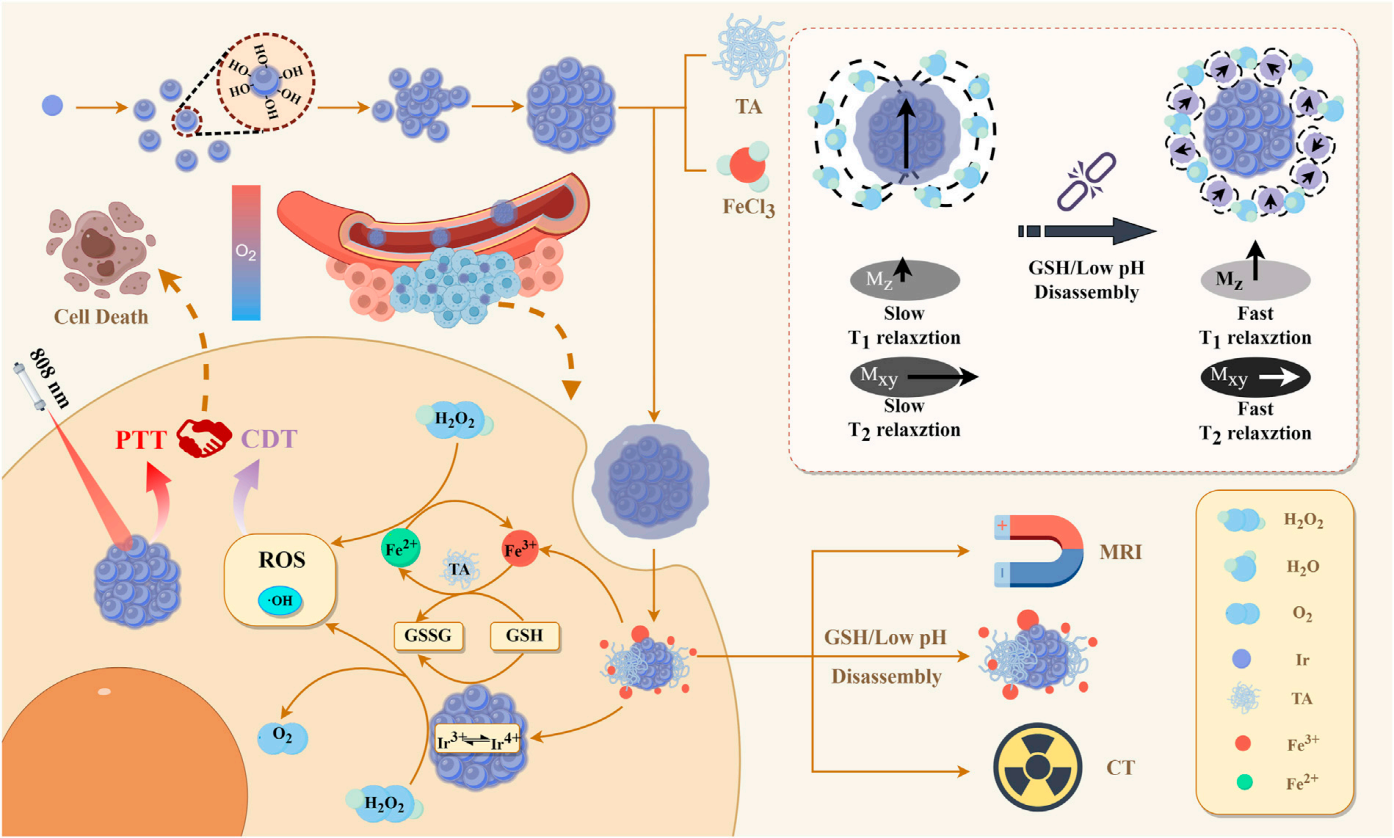
Illustration of the fabrication of IrO_x_@MPN for (T_1_/T_2_-weighted) MRI/CT multimodal imaging-guided CDT-PTT synergistic therapy.

**FIGURE 2:**
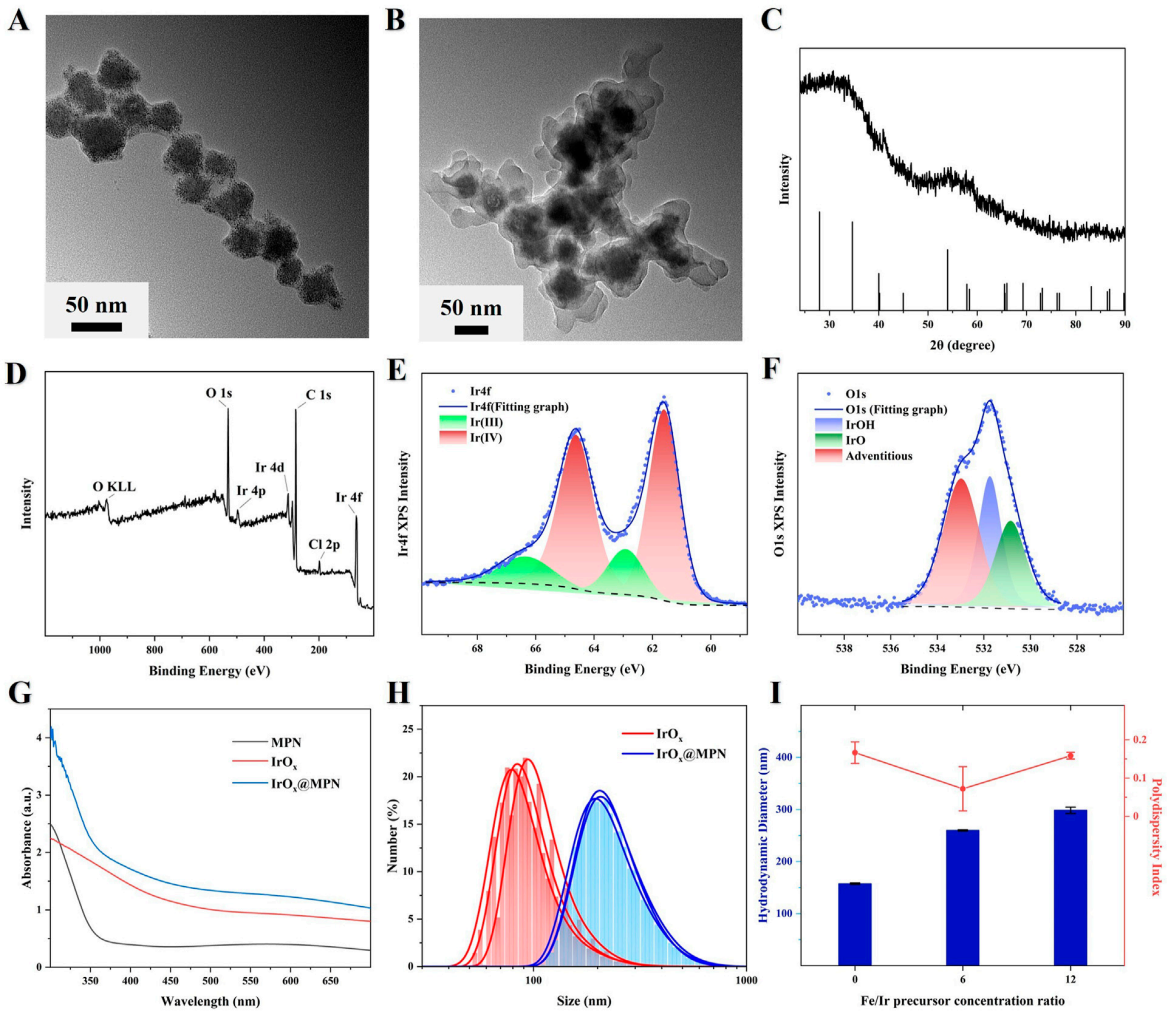
**(A,B)** TEM image of IrO_x_ and IrO_x_@MPN NPs. **(C)** XRD spectrum of IrOx NPs. **(D)** XPS spectrum of IrO_x_ NPs. **(E)** Ir 4f XPS spectrum of the IrO_x_ NPs. **(F)** O 1s XPS spectrum of the IrO_x_ NPs. **(G)** UV-Vis absorption spectrum of MPN, IrO_x_ and IrO_x_@MPN in aqueous solution. **(H)** Hydrodynamic diameter of IrO_x_ NPs and IrO_x_@MPN NPs. **(I)** Hydrodynamic diameter and polydispersity index with the different Fe/Ir precursor concentration ratios.

The synthesis process of IrO_x_@MPN is illustrated in Figure 1. We first synthesized multi-cage IrO_x_ NPs using a direct hydrothermal method in an alkaline environment by heating IrCl_3_ at 80°C for 13 h. In the alkaline solution of IrCl_3_, [Ir(OH)_6_]^3−^ initially forms, and upon heating to 80°C, iridium oxide nanoparticles gradually appear. Due to the thermal motion effect under heating conditions, smaller iridium oxide nanoparticles gradually self-aggregate into larger clusters, and after 13 h of continuous reaction, multi-cage IrO_x_ NPs are obtained. Subsequently, by adding TA and FeCl_3_ to the weakly alkaline solution of IrO_x_ NPs, we successfully synthesized IrO_x_@MPN using a one-step method. Initially, the multi-caged IrO_x_ nanoparticles with a spherical shape were synthesized through the direct thermal hydrolysis of IrCl3 in an alkaline environment, as exhibited in Figure 2A. The TEM images of IrO_x_@MPN clearly show a translucent shadow surrounding the IrO_x_ NPs, confirming the successful encapsulation of the MPN shell (Figure 2B). The XRD pattern of the synthesized IrO_x_ reveals a broad peak, which is attributed to the limitations of XRD for detecting small particles. (Figure 2C). The XPS spectrum indicates that IrO_x_ nanoparticles contain not only Ir and O elements but also foreign C and Cl elements from the raw materials (Figure 2D). The deconvolution of the XPS spectrum for the Ir 4f region of IrO_x_ reveals two binding peaks around 61.8 eV and 64.8 eV, corresponding to Ir^4+^, and peaks at approximately 62.4 eV and 65.6 eV, corresponding to Ir^3+^ (Figure 2E) (Wang D. et al., 2019). In the O 1s region, deconvoluted peaks at about 530.8 eV and 531.7 eV suggest the presence of iridium oxide and hydroxide, respectively (Figure 2F) (Zhang et al., 2013). Next, FeCl_3_ and TA were introduced to form the MPN layer on the surface of the IrO_x_ nanoparticles. The UV-Vis absorption spectrum of IrO_x_@MPN shows the merging of the characteristic absorption peaks of MPN and IrO_x_ nanoparticles (Figure 2G). Elemental mapping of IrO_x_@MPN reveals the core-shell architecture of the nanoplatform and shows a uniform distribution of Ir, Fe, and O elements (Supplementary Figure S1). This mapping provides additional confirmation that MPN has been effectively applied to the IrO_x_ surface, thanks to the adhesive properties of polyphenols. IrO_x_ and IrO_x_@MPN exhibit good dispersion in water, with hydrodynamic sizes of approximately 157.4 nm and 259.8 nm, respectively (Figure 2H). The larger size observed in the Dynamic Light Scattering (DLS) measurements, compared to the sizes seen in SEM and TEM images, is likely due to swelling or slight aggregation of IrO_x_ and IrO_x_@MPN in solution. Additionally, by controlling the Fe/Ir precursor ratio, the two-step synthesis strategy allows the thickness of the MPN layer to be manipulated, thereby influencing the structure, morphology, and Fe content of the nanoplatform (Figure 2I; Supplementary Figure S2).

**FIGURE 3:**
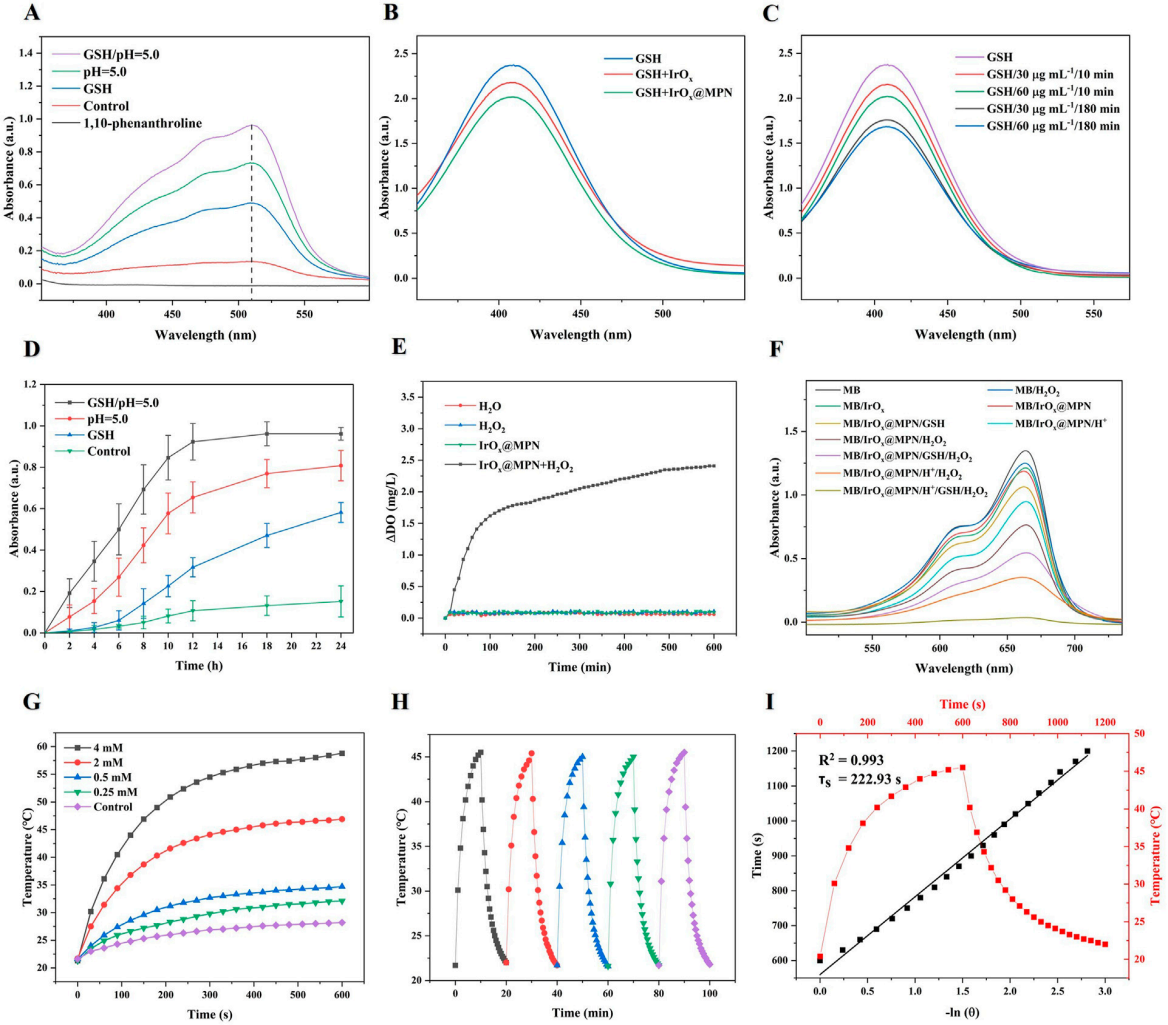
**(A)** UV–Vis spectra of pure 1,10-phenanthroline and the mixture of 1,10-phenanthroline and IrO_x_@MPN with or without GSH or H^+^. **(B)** GSH depletion (1 mM) under the reduction of IrO_x_ and IrO_x_@MPN aqueous solution (50 μg/mL). **(C)** GSH depletion (1 mM) under the reduction of different concentrations (30, 60 μg/mL) and different times (10, 180 min) of IrO_x_@MPN. **(D)** Time-dependent absorbance at 510 nm for the detection of Fe (III) released using 1,10-phenanthroline under different conditions. **(E)** O_2_ generation of IrO_x_@MPN NPs with H_2_O_2_ (100 μM). **(F)** UV–Vis absorption spectra of MB solution treated with different groups. **(G)** Temperature variation of IrO_x_@MPN NPs aqueous solutions with different concentrations over 10 min irradiation (1.0 W/cm^2^). **(H)** Heating curve of IrO_x_@MPN NPs aqueous solution (0.5 mg/mL) for five cycles under irradiation of 808 nm laser (1.0 W/cm^2^). **(I)** The time constant for heat transfer from the system was determined to be τ_s_ = 222.93 s by applying the linear time data from the cooling period (after 10 min) versus negative natural logarithm of the driving force temperature obtained from the cooling stage.

Reports indicate that MPN is prone to decomposition when exposed to GSH or in acidic environments (Liu et al., 2023). Due to the formation of a stable orange-colored complex between 1,10-phenanthroline and Fe^2+^, with an absorbance peak at 508 nm, it can be utilized for the detection of Fe^2+^ generated from the reduction of Fe^3+^ (Liu et al., 2023). Therefore, we employed this method to investigate the GSH and pH-responsive disassembly behavior of IrO_x_@MPN by measuring the release of Fe^2+^. As depicted in Figure 3A, the group treated with IrO_x_@MPN alone showed no significant UV absorption peak. However, a slight increase in absorbance at 508 nm was detected for IrO_x_@MPN after exposure to GSH. As expected, a significant increase in absorbance intensity at 508 nm was observed when exposed to both acidic conditions and GSH, indicating the significant release of Fe^2+^ from the MPN. The above results confirm the dual-responsive disassembly behavior of the MPN shell. This phenomenon is mainly because the phenolic hydroxyl groups of TA form strong coordination interactions with metal ions under neutral or alkaline conditions, whereas acidic conditions induce protonation of the hydroxyl groups of TA, resulting in weakened metal-phenol coordination (Liu et al., 2023). Additionally, the release of Fe³⁺ from IrO_x_@MPN in the presence of GSH is linked to the reduced coordination bonds, which occur because Fe³⁺ is reduced. It is important to note that, unlike traditional metal ion-coated nanoparticles, the dual-responsive decomposition of MPN maximizes the utilization efficiency of metal ions (Wang et al., 2022). Subsequently, the enclosed IrO_x_ are exposed, thereby unleashing the multi-enzyme mimetic functionalities, and providing another reaction pathway for CDT. DTNB, with a maximum absorbance of 408 nm, was used as an indicator to assess the GSH depletion ability of IrO_x_ and IrO_x_@MPN. Figure 3B demonstrates that the characteristic absorbance peak of GSH + DTNB at 408 nm is prominently observed. In contrast, the absorbance at this wavelength decreases in the IrO_x_@MPN-treated groups, signifying effective GSH consumption by IrO_x_@MPN. Over time, there is a marked reduction in the absorption intensity at 408 nm. This decline occurs because GSH reduces Fe^3+^, and simultaneously, the iridium ions in IrO_x_@MPN deplete GSH through a cyclic valence change mechanism (Figure 3C). Subsequently, we detected the release of Fe^3+^ from IrO_x_@MPN under different conditions using 1,10-phenanthroline. It was observed that both GSH and acidic conditions led to the degradation of the MPN shell and the release of Fe^3+^. Moreover, under the combined effect of both conditions, the release rate of Fe^3+^ from the MPN shell significantly increased, demonstrating the dual-responsive degradation capability of the MPN shell to GSH and acidic conditions (Figure 3D). Furthermore, TEM images of IrO_x_@MPN treated under GSH and acidic conditions confirm the degradation of the MPN shell (Supplementary Figure S2). The significant GSH consumption by IrO_x_@MPN helps to reduce the quenching of ROS effectively. The synthesized IrO_x_@MPN demonstrated catalase-like activity, as evidenced by the detection of dissolved oxygen (DO) following the addition of H_2_O_2_ to the IrO_x_@MPN solution (Figure 3E). To assess the capability of IrO_x_@MPN for ·OH generation, methylene blue (MB) was used as an indicator, since ·OH can degrade MB. Figure 3F illustrates the variations in MB degradation across different experimental groups. Compared with the individual IrO_x_@MPN with H_2_O_2_, the solution containing IrO_x_@MPN + H^+^+GSH + H_2_O_2_ exhibits the most pronounced decrease in the absorption peak at 664 nm. This suggests that the ability of IrO_x_@MPN to generate ·OH is significantly enhanced under GSH and acidic conditions. The above experimental results indicate the remarkable GSH depletion ability and the high Fenton reaction activity of IrO_x_@MPN through acid and GSH response. Furthermore, owing to its distinctive multi-caged structure, IrO_x_ exhibits a larger specific surface area, leading to a higher photothermal conversion efficiency of IrO_x_@MPN. The photothermal performance of IrO_x_@MPN was evaluated by recording the temperature changes in its aqueous solutions, which were subjected to 808 nm laser irradiation, using a digital thermometer. The results revealed that the photothermal effect increased with concentration (Figure 3G). Specifically, the temperature of a 4 mM IrO_x_@MPN solution rose to 58.8°C within 10 min of exposure to a 1.0 W/cm^2^ laser. Additionally, IrO_x_@MPN, which demonstrates strong thermal stability (Figure 3H), has a photothermal conversion efficiency of 60.78% (Figure 3I), which is higher than the reported iridium-based nanomaterials and the majority of photothermal nanomaterials (Zhen et al., 2020b; Li et al., 2017; Ji et al., 2016; Yong et al., 2015).

**FIGURE 4:**
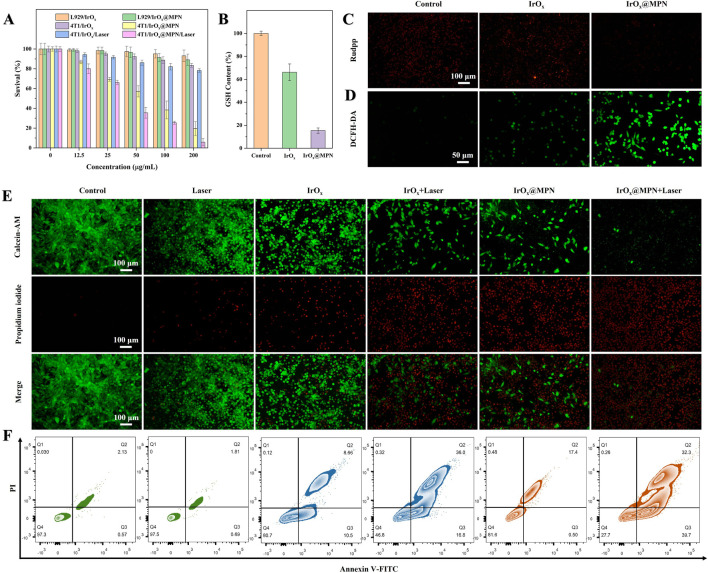
**(A)** Survival rate of different cells incubated with different groups for 24 h **(B)** GSH content of 4T1 cells with or without incubation with IrO_x_ and IrO_x_@MPN. **(C)** Fluorescent images of 4T1 cells in various groups and stained by Rudpp. **(D)** Fluorescent images of 4T1 cells in various groups and stained by DCFH-DA. **(E)** Calcein-AM/PI staining images of 4T1 cells after different treatments. **(F)** Flow cytometry analysis on the apoptosis levels of 4T1 cells treated with different formulations.

Since effective cellular uptake is essential for achieving a strong therapeutic effect, IrO_x_@MPN was labeled with Fluorescein Isothiocyanate Isomer I (FITC) to track its intracellular absorption. As depicted in Supplementary Figure S3, a distinct green fluorescence was observed in 4T1 cells, indicating successful uptake of the nanomaterials. At the 1 h observation point, the cellular uptake of IrO_x_@MPN reached a plateau. We observed a comparable cellular uptake of IrO_x_@MPN to that of standalone IrO_x_, indicating that the MPN shell coating does not affect cellular uptake. The CCK-8 assay was employed to evaluate the cytotoxicity of IrO_x_@MPN, as illustrated in Figure 4A. IrO_x_@MPN displayed no noticeable toxicity to mouse fibroblasts (L929 cells), even at elevated concentrations. At 200 μg/mL, cell viability remained at 89.0%, indicating that IrO_x_@MPN exhibits favorable biocompatibility. Due to the inhibitory effect of excessive GSH in the TME on the therapeutic efficacy of ROS generation during CDT, we employed a reduced GSH assay kit to further evaluate the GSH consumption capability of IrO_x_@MPN. This process was crucial in understanding the potential of IrO_x_@MPN to overcome the GSH-mediated resistance in CDT. Figure 4B illustrates that GSH levels in the IrO_x_@MPN group were significantly reduced relative to both the control group and the IrO_x_ group. This result corroborates the DTNB degradation assay findings, highlighting the capability of IrO_x_@MPN to effectively reduce intracellular GSH levels. In 4T1 cells, IrO_x_@MPN could efficiently convert H_2_O_2_ into O_2_, resulting in almost complete quenching of the fluorescence of the oxygen probe [Ru (DPP)_3_]Cl_2_ (Rudpp) in comparison to cells treated with IrO_x_ or PBS (Figure 4C). 2′,7′-dichlorofluorescin diacetate (DCFH-DA) is employed to measure the levels of ROS level in cells. Figure 4D demonstrates that 4T1 cells treated with IrO_x_@MPN displayed notably more intense green fluorescence compared to both the control and IrO_x_ groups. This suggests that the increase in intracellular oxygen level will enhance the production of ROS in cancer cells to some extent. It may be attributed to the differential GSH consumption capacity between IrO_x_ and IrO_x_@MPN, along with the presence of Fe^2+^ in IrO_x_@MPN. Although both IrO_x_ and IrO_x_@MPN can deplete GSH and generate ROS, the enhanced GSH consumption capability of IrO_x_@MPN is more effective in disrupting the intracellular redox balance. Additionally, IrO_x_@MPN has the ability to induce ROS production through dual pathways simultaneously. Consequently, IrO_x_@MPN exhibits a stronger CDT effect compared with IrO_x_. This result was confirmed in the dual staining experiment with calcein-AM and PI (Figure 4E). Without laser irradiation, the IrO_x_@MPN group showed a considerable increase in cell death compared to both the control and IrO_x_ groups. With 808 nm laser exposure, the combination of CDT and PTT resulted in almost complete cell death, underscoring the immense potential of IrO_x_@MPN as an effective strategy for CDT/PTT. Additionally, cell apoptosis was analyzed by employing the Annexin V-FITC and PI dual staining technique. Flow cytometry was employed to confirm the enhanced therapeutic efficacy of the combined CDT/PTT approach (Figure 4F). Initially, without laser irradiation, apoptosis rates in cells exposed to IrO_x_@MPN were notably higher (19.16%) compared to the control group (2.7%). After 10 min of 808 nm laser exposure, the IrO_x_@MPN/Laser group exhibited a marked increase in 4T1 cell apoptosis (72.0%), significantly surpassing the rates observed in both the control group (2.5%) and the IrO_x_ group (52.8%). Under the dual-responsive conditions of acidity and GSH, IrO_x_@MPN achieves more effective CDT by disrupting the GSH-mediated cellular ADS and inducing ROS production through dual pathways. When combined with PTT, the apoptosis of cancer cells could be effectively achieved.

**FIGURE 5:**
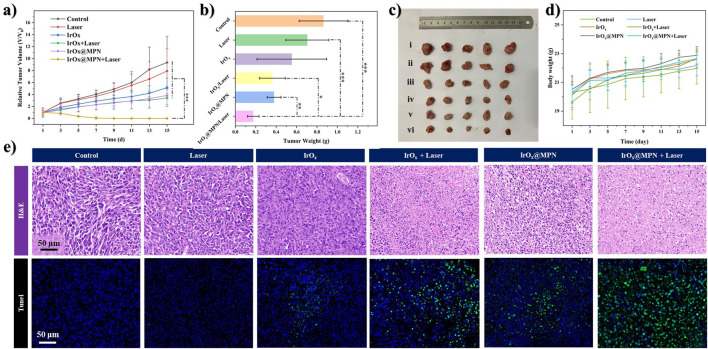
**(A)** Relative tumor growth trend curves of mice treated with different formulations (n = 5, *p < 0.05, **p < 0.01, ***p < 0.001). **(B)** The average tumor mass in mice sacrificed after 14 days observation. *p < 0.05, **p < 0.01, ***p < 0.001. **(C)** The digital photos of excised tumors in different groups after 14 days of treatment (i. Control; ii. Laser; iii. IrO_x_; iv. IrO_x_ + Laser; v. IrO_x_@MPN; vi. IrO_x_@MPN + Laser). **(D)** Variation of body weights of tumor-bearing mice with different treatments. **(E)** 4T1 tumor tissues stained with H&E, TUNEL (cell nuclei were stained with DAPI) in different groups.

Based on the promising *in vitro* results, we developed 4T1 tumor models in mice to assess the effectiveness of IrO_x_@MPN in inhibiting tumor growth *in vivo*. The mice were randomly assigned to six different groups: Control, Laser, IrO_x_ (single-pathway CDT), IrO_x_ + Laser (single-pathway CDT/PTT), IrO_x_@MPN (dual-pathway CDT), and IrO_x_@MPN + Laser (dual-pathway CDT/PTT) 24 h after intravenous injection. In the laser-treated groups, tumors were exposed to 808 nm laser irradiation for 10 min at a power density of 1.0 W/cm^2^. Throughout the 15-day treatment period, tumor size and body weight were measured every 2 days (Figure 5A). The tumors in the IrO_x_@MPN + Laser group showed significant suppression, whereas tumors in the other groups did not exhibit similar effects (Figures 5B–D). To assess the antitumor activity of IrO_x_@MPN at a tissue level, tumor sections were subjected to histological staining (Figure 5E). The H&E staining results revealed that tumors treated with IrO_x_@MPN + Laser exhibited the most pronounced karyolysis and plasmatorrhexis. Furthermore, the TUNEL staining images of tumor sections from the IrOx@MPN + Laser group displayed the most intense green fluorescence, indicating a significant degree of tumor inhibition. The above results proved that IrO_x_@MPN showed a significant therapeutic effect in the combination therapy of CDT and PTT by enhancing GSH consumption and dual-pathway ROS generation. Throughout the treatment period, no significant variations in body weight were detected among the experimental and control groups. (Figure 5D). Additionally, no significant tissue damage was observed in the primary organs of mice treated with IrO_x_@MPN (Supplementary Figure S4). Hematology analysis showed no significant difference between IrO_x_@MPN and the control group (Supplementary Figure S5), further confirming the satisfactory biosafety of the IrO_x_@MPN nanoplatform.

**FIGURE 6:**
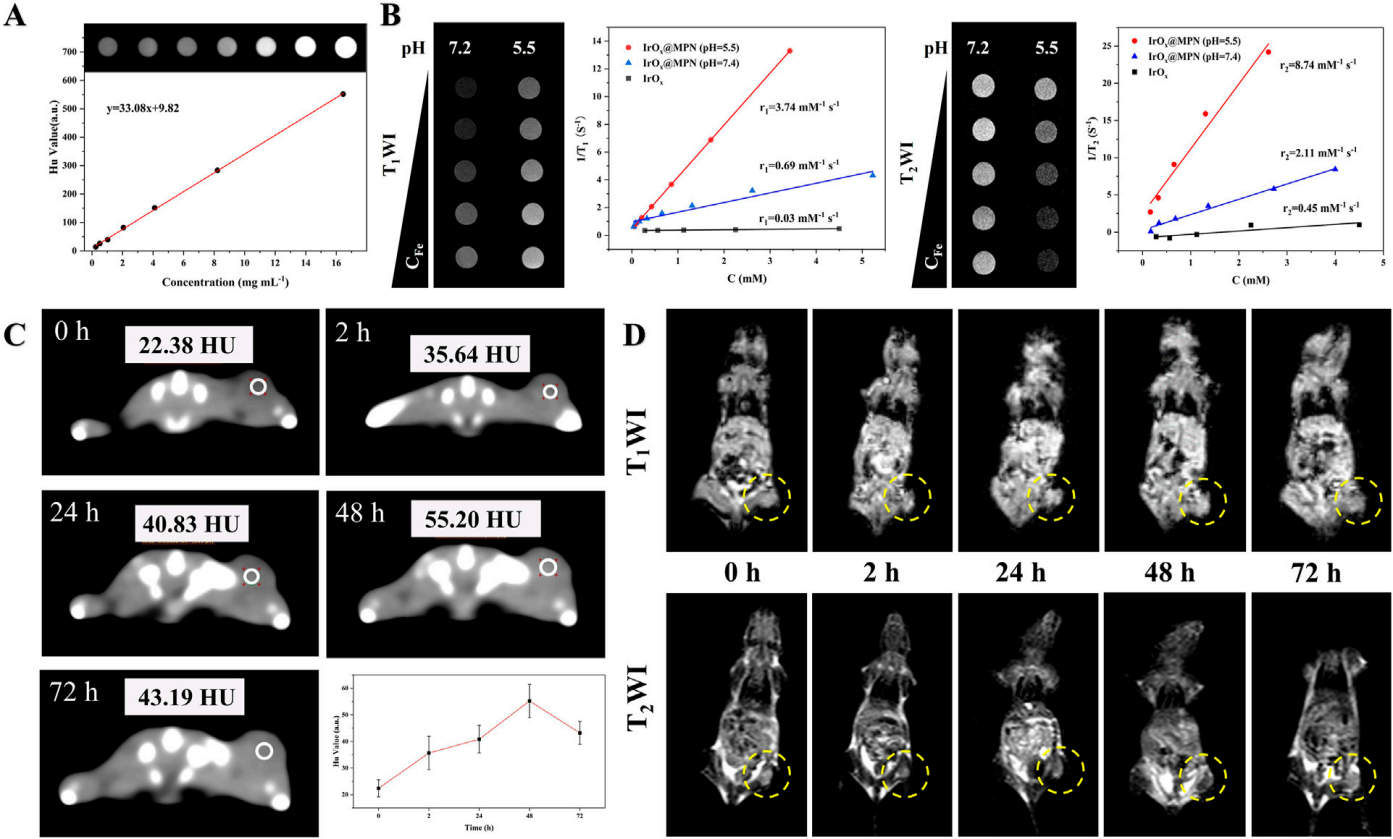
Multi-model imaging effects of IrO_x_@MPN NPs. **(A)** *In vitro* computed tomography signals of IrO_x_@MPN NPs with different concentrations. **(B)** Plots of 1/T_1_ and 1/T_2_ over the concentrations of Fe from IrO_x_@MPN NPs. **(C)** *In vivo* computed tomography images of Balb/C mice pre- and post- intravenously injected with 100 μL of IrO_x_@MPN (3 mg/mL in saline). **(D)** *In vivo* T_1_/T_2_ weighted MRI photos of tumor-bearing mouse after intravenous injection of IrO_x_@MPN NPs (100 μL, 2 mg/mL in saline) at various time intervals.

Considering the excellent X-ray absorption capability of Ir, we investigated the *in vitro* CT imaging capability of IrO_x_@MPN. As illustrated in Figure 6A, a strong linear relationship was observed between Hounsfield units (HU) and Ir concentration, with a calculated X-ray attenuation coefficient of 33.08 HU·mL/mg for IrO_x_@MPN, which is higher than clinical iodine-based contrast agent (16.4 HU·mL/mg). Moreover, since Fe^2+^ and Fe^3+^ possess multiple unpaired electrons, IrO_x_@MPN can serve as an MRI contrast agent theoretically. As shown in Figure 6B, the T_1_ and T_2_ signal of IrO_x_@MPN gradually increased with increasing Fe concentration. The values of longitudinal relaxivity (r_1_) and transverse relaxivity (r_2_) values were measured at 0.69 mM/s and 2.11 mM/s, respectively. At pH 5.5, the signal contrast on both T_1_WI and T_2_WI of IrO_x_@MPN is enhanced, particularly in T_2_WI. The r_1_ and r_2_ relaxation rates increase to 3.74 mM/s and 8.74 mM/s, respectively. To further assess its potential as an *in vivo* multimodal CT and MR imaging contrast agent, we intravenously injected IrO_x_@MPN into 4T1 tumor-bearing mice and performed CT and MRI scans at several intervals (0 h, 2 h, 24 h, 48 h). After intravenous administration, effective accumulation of IrO_x_@MPN at the tumor site was noted, attributed to the EPR effect. CT images revealed significant enhancement in visual contrast at 24 h after injection, reaching a peak at 48 h, with an increase from 22.38 HU to 55.20 HU (Figure 6C). For T_1_WI and T_2_WI, distinct differences in signal intensity were observed between the tumor region and surrounding normal tissues, with the optimal effect reached at 48 h post-injection (Figure 6D). These results demonstrate that IrO_x_@MPN, with its exceptional capabilities, is an excellent candidate for use as a contrast agent in both CT and MRI (T_1_/T_2_) multimodal imaging.

## 4 Conclusion

In summary, we constructed a versatile nanoplatform (IrO_x_@MPN) consisting of an IrO_x_ core and self-assembled MPN based on Fe^3+^ and TA. This nanoplatform could respond to GSH and pH in the TME. This dual responsiveness sets the foundation for a powerful CDT, showcasing its potential in cancer treatment. The MPN shell composed of Fe^3+^ and TA, could undergo responsive disassembly in the TME. The valence state cycling of Fe^3+^/Fe^2+^ and Ir^4+^/Ir^3+^ through GSH consumption disrupts the ADS, leading to a redox imbalance and significantly enhancing the Fe- and Ir-mediated Fenton and Fenton-like reaction, thus achieving a remarkable dual-pathway CDT. Simultaneously, the high photothermal conversion efficiency of iridium-based nanomaterials and the unique multi-cage structure of IrO_x_ endow IrO_x_@MPN with a powerful PTT capability. Additionally, the presence of Ir and iron ions in IrO_x_@MPN grants it excellent CT and MRI (T_1_/T_2_) multimodal imaging capabilities, making it a promising diagnostic tool for tumor diagnosis. As a result, IrO_x_@MPN, as an easy-to-synthesize nanoplatform, achieves dual-pathways CDT combined PTT on the basis of degradation in response to acidic microenvironment and GSH. It also possesses stable multimodal imaging capabilities, holding immense potential in early cancer diagnosis, treatment, and therapeutic response monitoring. This study provides a feasible strategy for constructing an integrated nanoplatform with dual responsiveness for the diagnosis and treatment of TME, showing promising potential for future development.

## Data Availability

The original contributions presented in the study are included in the article/Supplementary Material, further inquiries can be directed to the corresponding authors.
