# Supplementary figures and images for: Dual-responsive nanoplatform for integrated cancer diagnosis and therapy: Unleashing the power of tumor microenvironment
Source: Front Chem. 2024 Sep 26;12:1475131. doi: 10.3389/fchem.2024.1475131 (PMC11464441; doi:10.3389/fchem.2024.1475131)

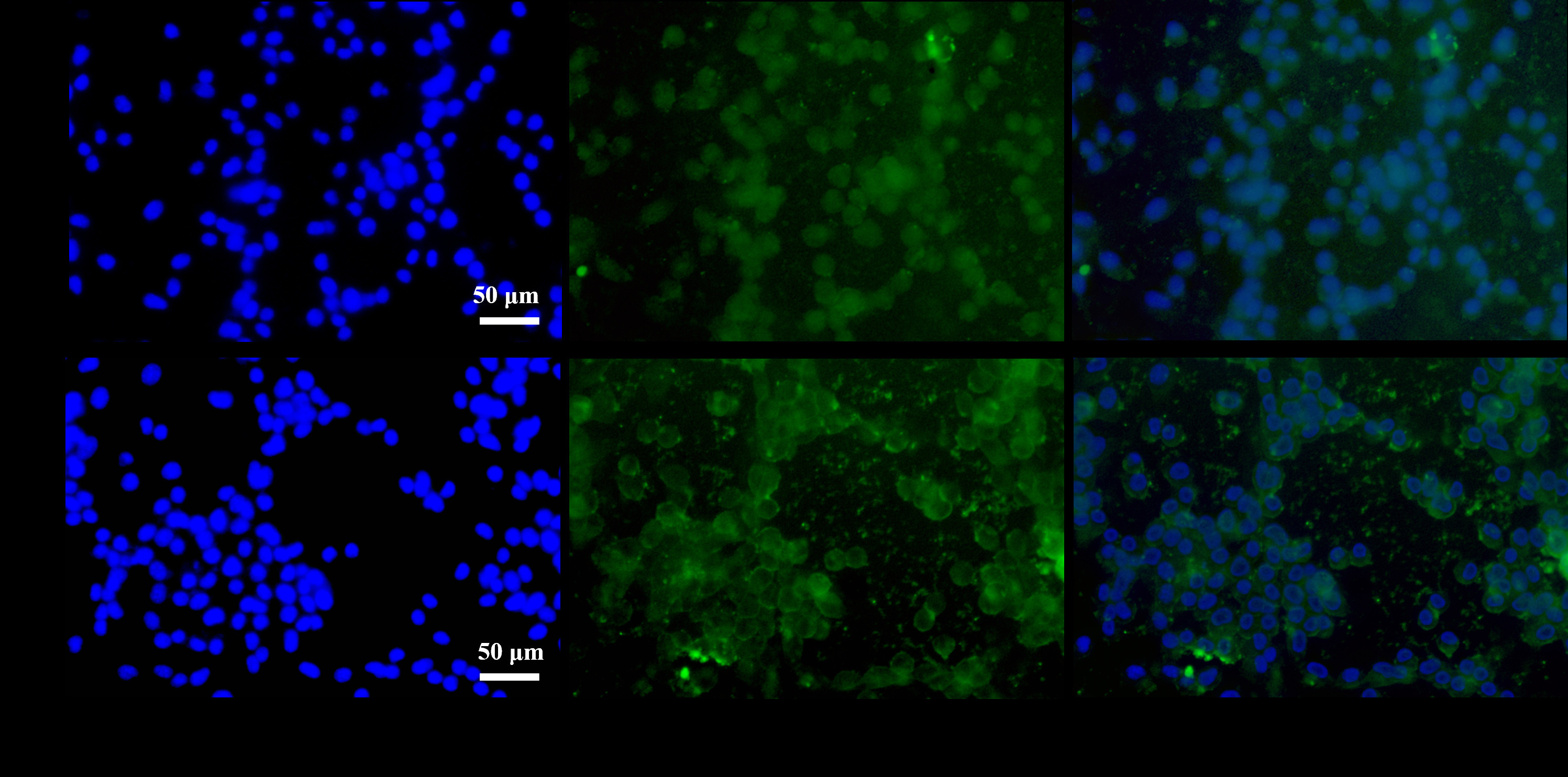

Supplement: Supplementary file 1 [file Image3.JPEG]

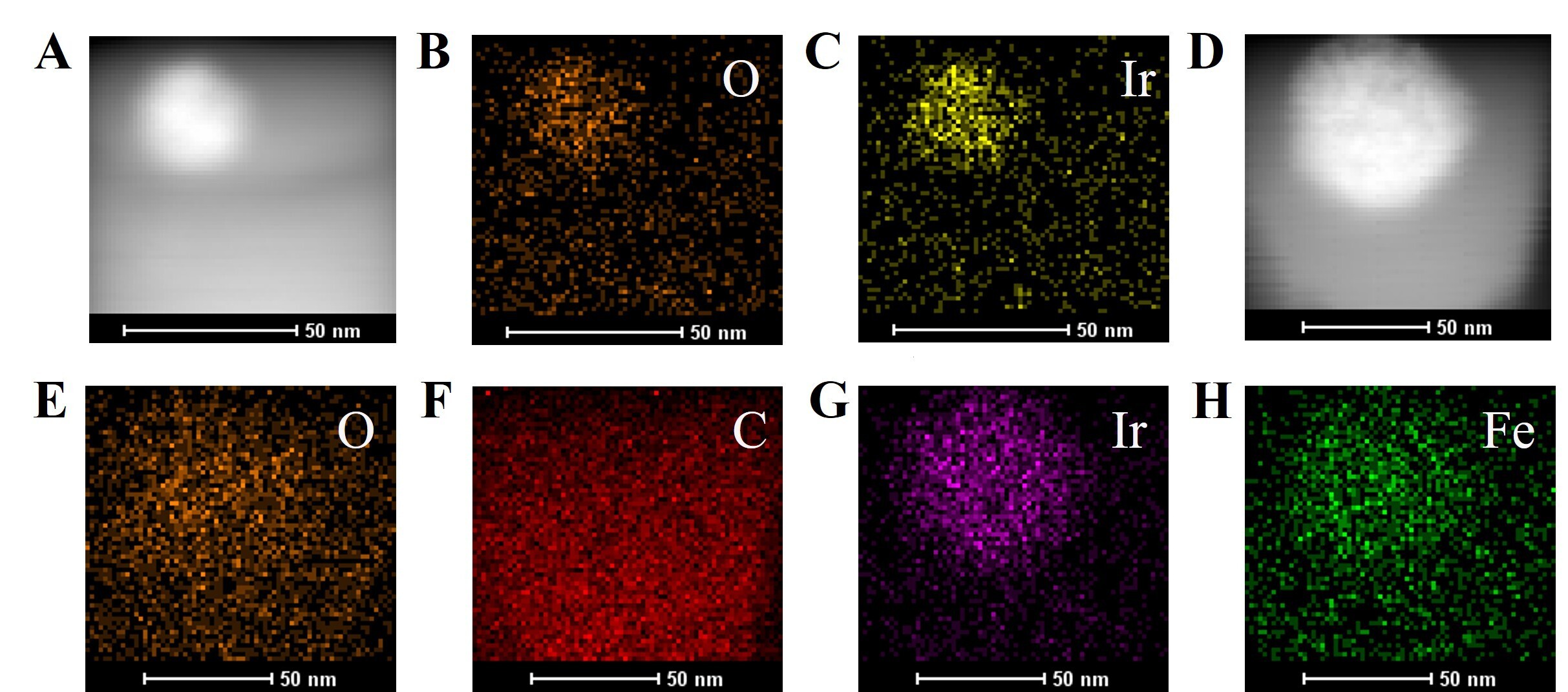

Supplement: Supplementary file 2 [file Image1.JPEG]

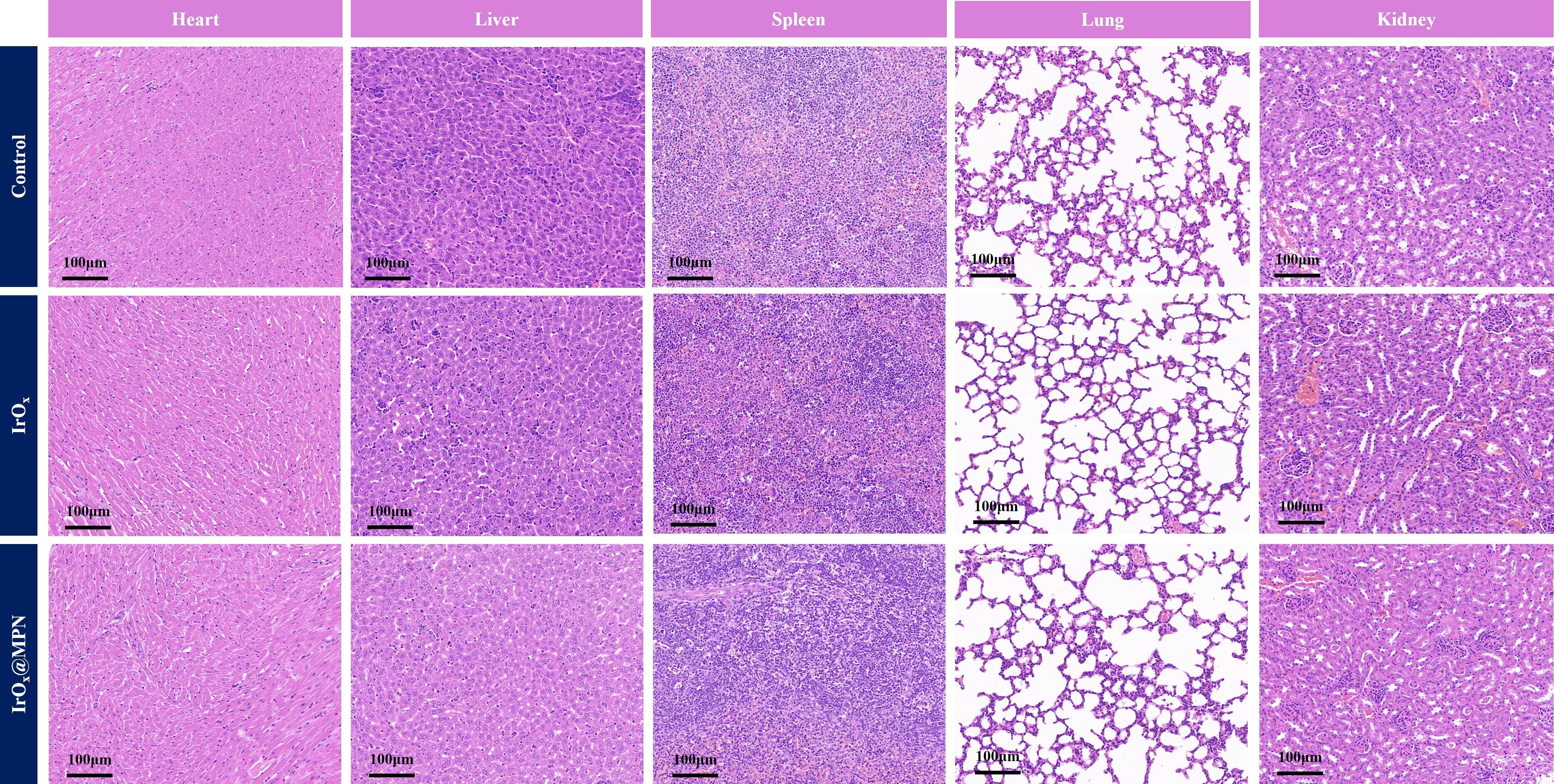

Supplement: Supplementary file 3 [file Image4.JPEG]

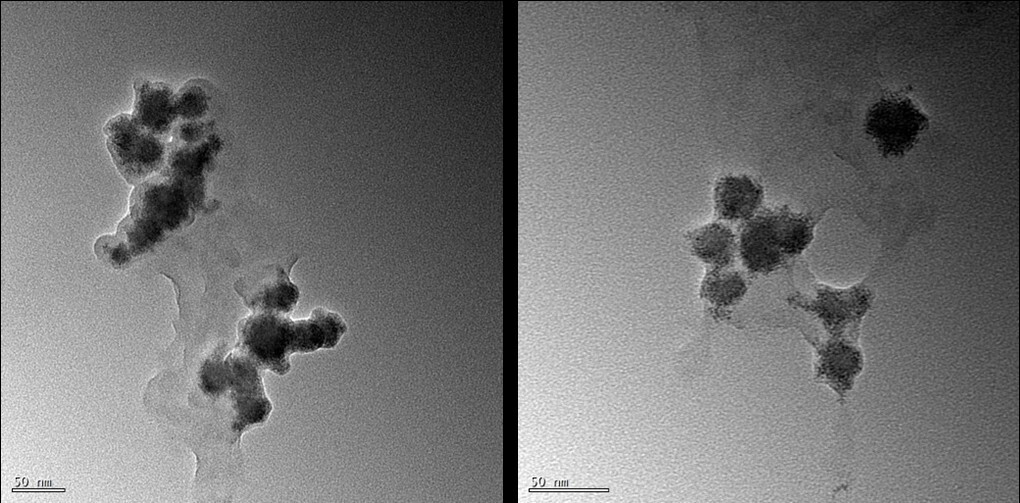

Supplement: Supplementary file 4 [file Image2.JPEG]

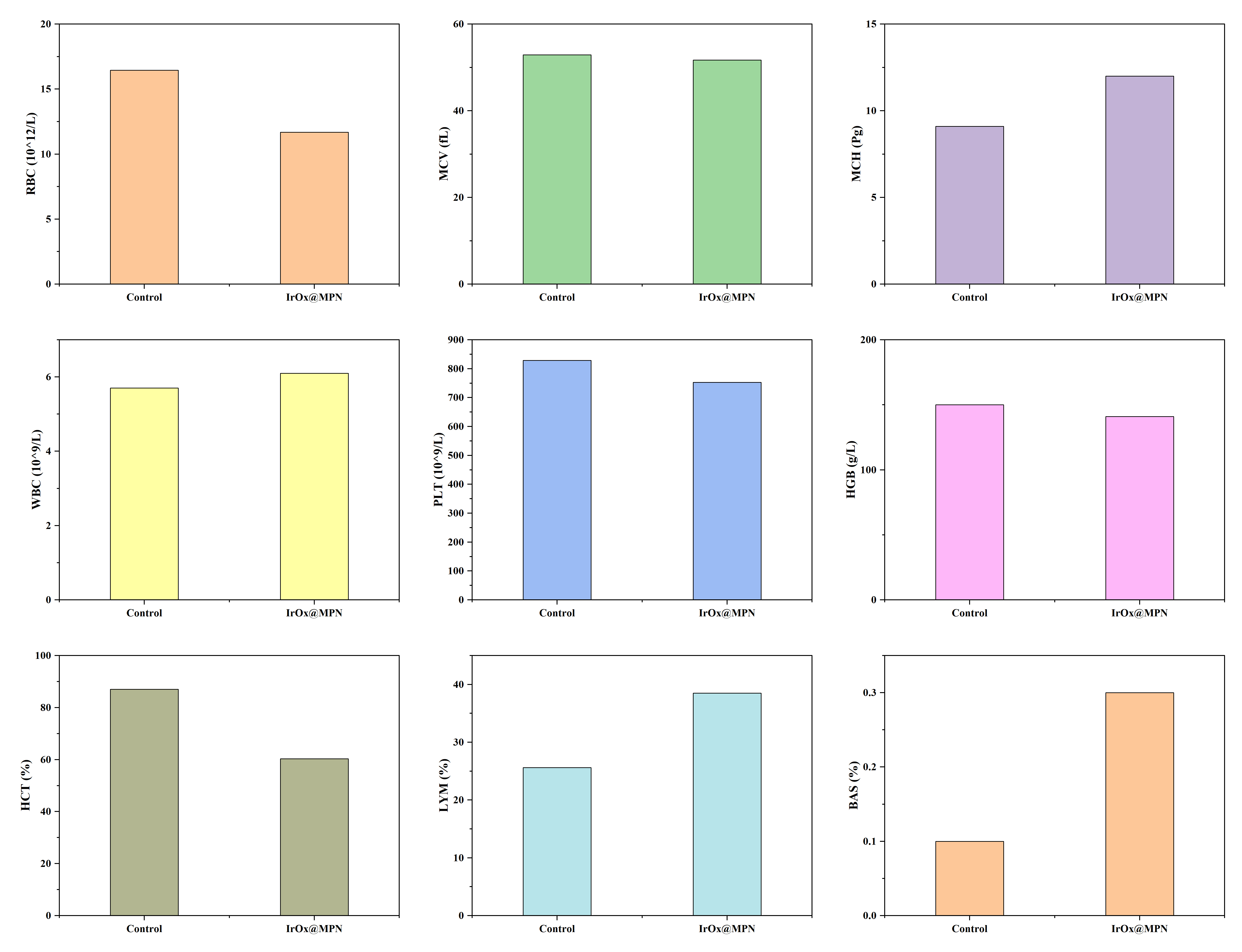

Supplement: Supplementary file 5 [file Image5.JPEG]

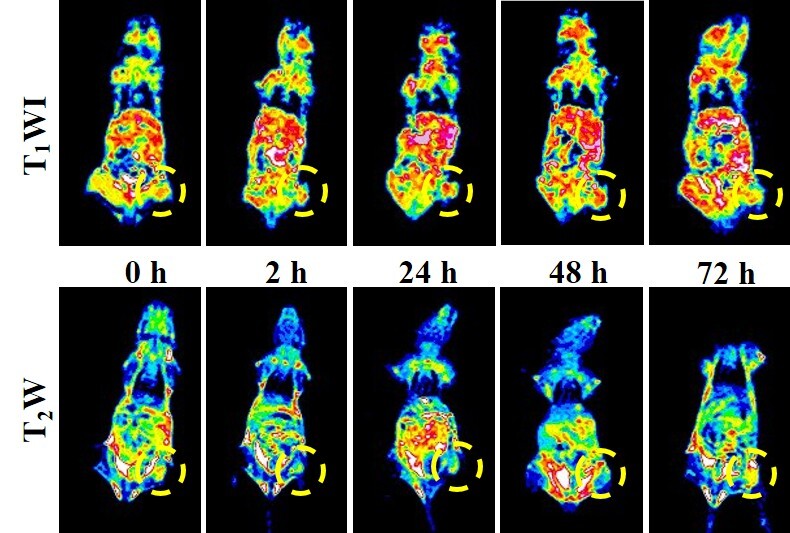

Supplement: Supplementary file 7 [file Image6.JPEG]
