# Supplementary material for: Dual-responsive nanoplatform for integrated cancer diagnosis and therapy: Unleashing the power of tumor microenvironment
Source: Front Chem. 2024 Sep 26;12:1475131. doi: 10.3389/fchem.2024.1475131 (PMC11464441; doi:10.3389/fchem.2024.1475131)
Supplement: Supplementary file 6 [file DataSheet1.pdf]

Dual-Responsive Nanoplatform for Integrated Cancer Diagnosis and Therapy: Unleashing the Power of Tumor Microenvironment

Rui Ma[1], Peng Zhang[1], Xiuying Chen[1], Mengdi Zhang[1], Qinghe Han[1,*], Qinghai Yuan[1,*]

[1] Department of Radiology, The Second Hospital of Jilin University, Changchun 130041, China

*** Correspondence:**
hanqinghe@mails.jlu.edu.cn (Qinghe Han); yuanqinghai123@sina.com (Qinghai Yuan).

Supplementary information

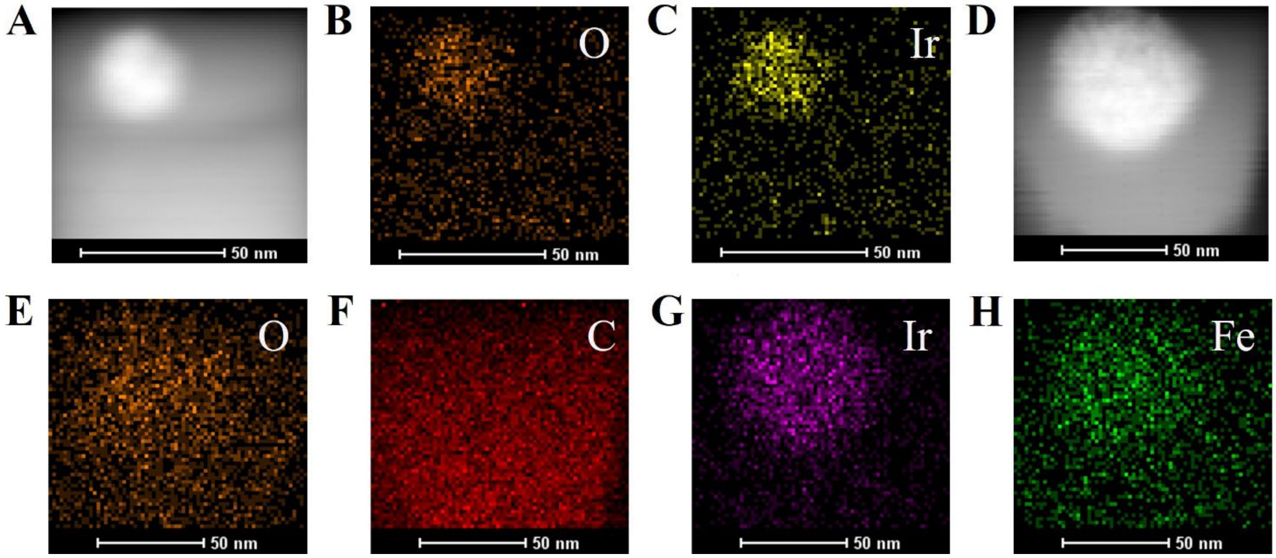

Figure S1. (A-C) Elemental mapping of IrO_x. (D-H) TEM mapping of IrO_x@MPN.

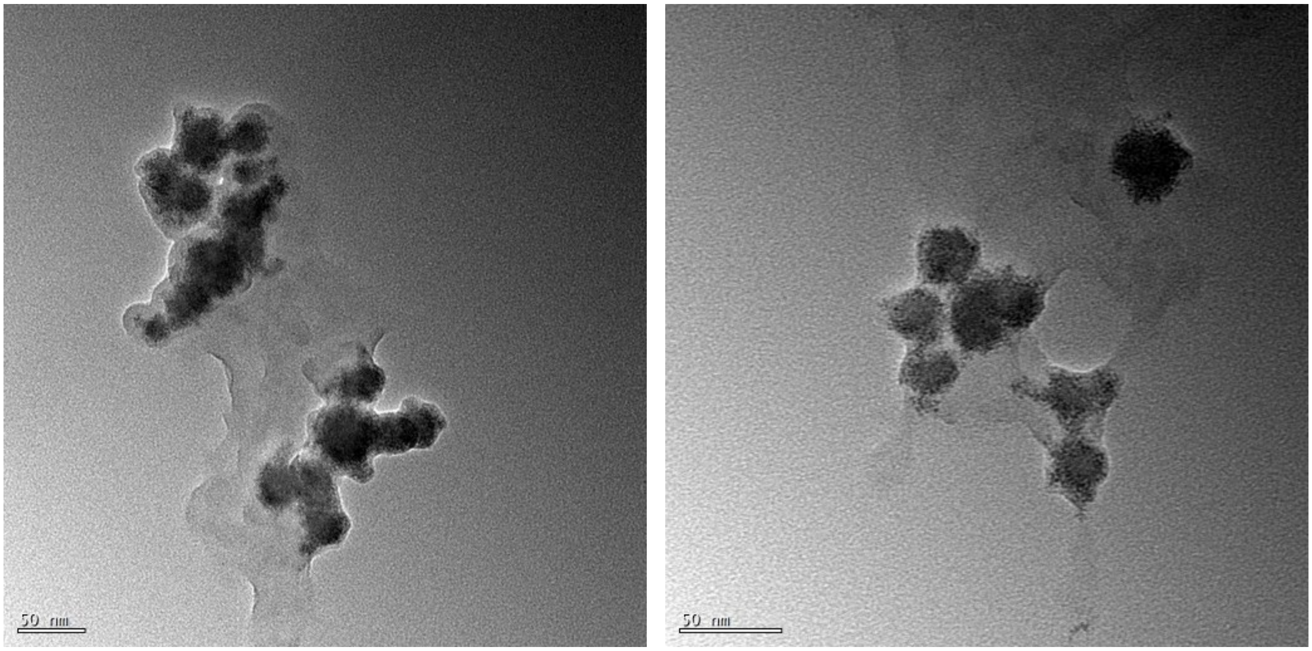

Figure S2. TEM images of IrOx@MPN treated under GSH and acidic conditions (Scale bar: 50 nm).

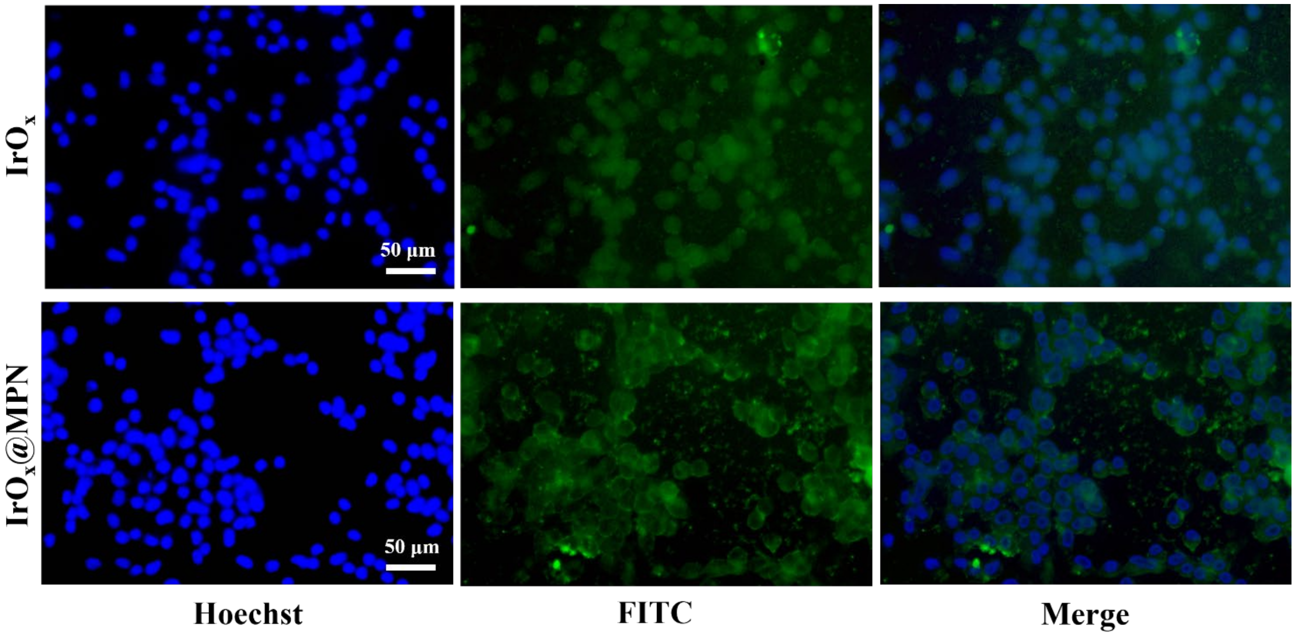

Figure S3. Fluorescent images of 4T1 cells in different groups stained by Hoechst and FITC (1 h).

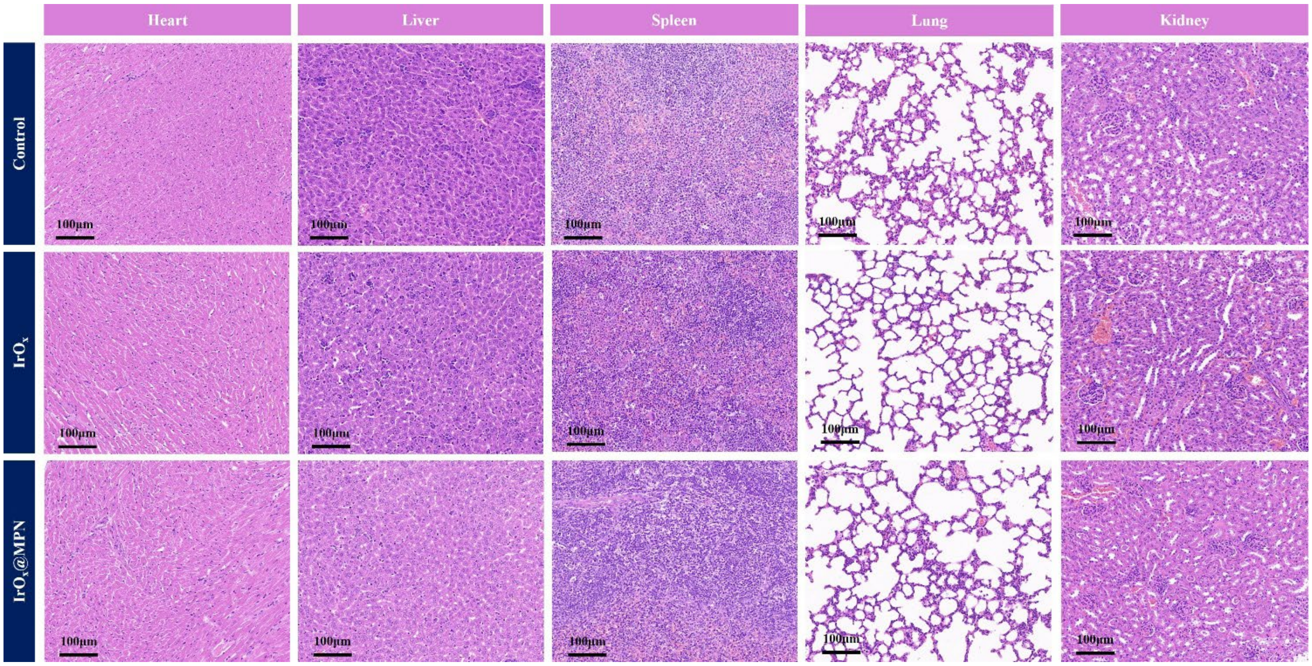

Figure S4. Representative H&E staining images of major organs collected from mice receiving the indicated treatments.

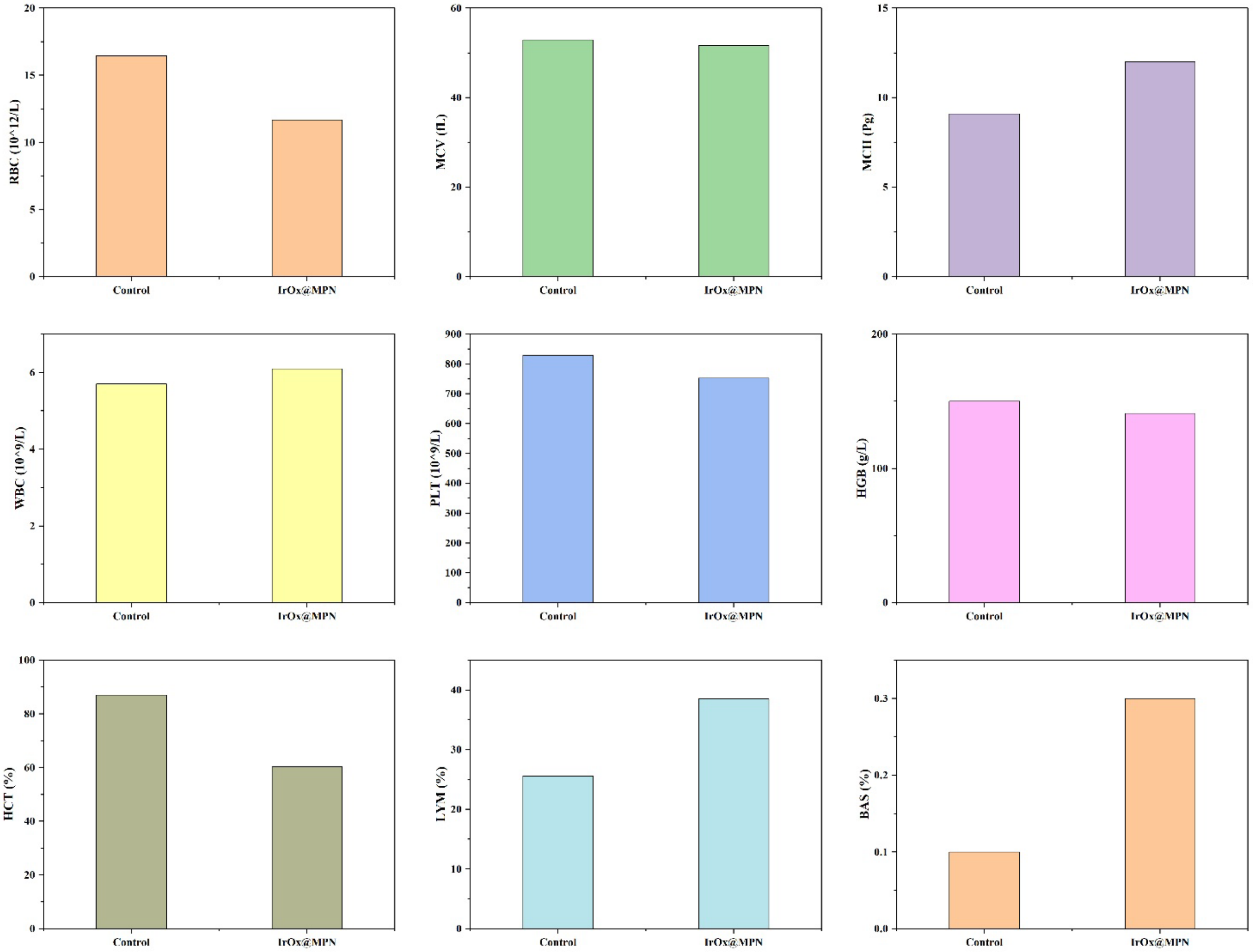

Figure S5. Hematology analysis of blood of mice with or without the intravenous injection of IrO_x@MPN after 30 days.

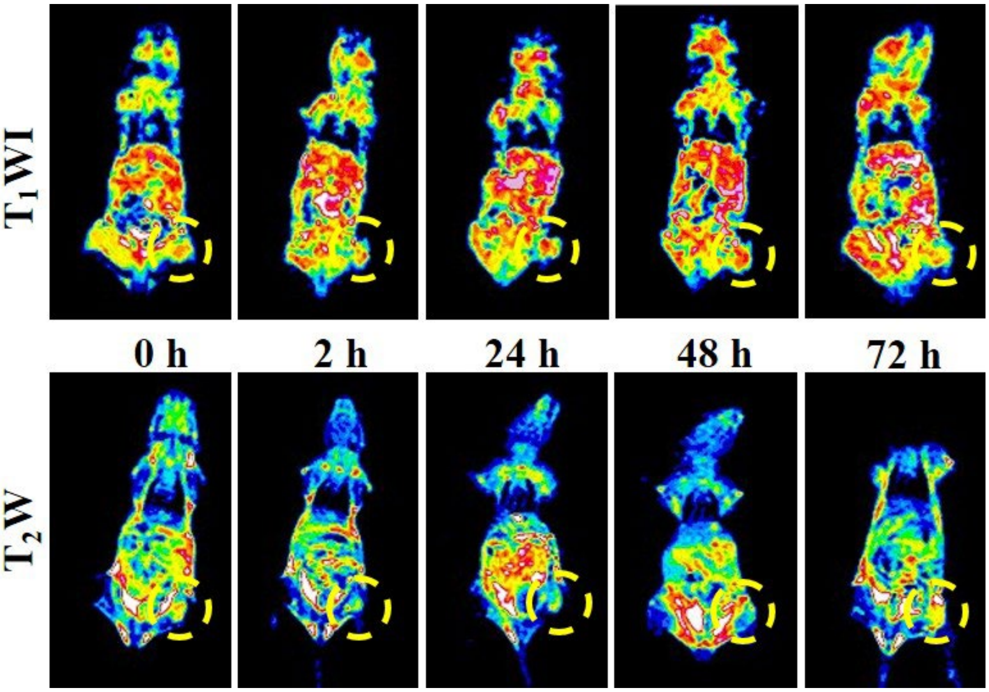

Figure S6. Pseudocolor images generated from T_1/T_2-weighted MRI scans at different time intervals after intravenous injection of IrO_x@MPN NPs (100 μL, 2 mg/mL in saline) in tumor-bearing mice.
